# Head-To-Head Comparison of PET and Perfusion Weighted MRI Techniques to Distinguish Treatment Related Abnormalities from Tumor Progression in Glioma

**DOI:** 10.3390/cancers15092631

**Published:** 2023-05-05

**Authors:** Dylan Henssen, Lars Leijten, Frederick J. A. Meijer, Anja van der Kolk, Anne I. J. Arens, Mark ter Laan, Robert J. Smeenk, Anja Gijtenbeek, Elsmarieke M. van de Giessen, Nelleke Tolboom, Daniela E. Oprea-Lager, Marion Smits, James Nagarajah

**Affiliations:** 1 Department of Medical Imaging, Radboud University Medical Center, 6525 GA Nijmegen, The Netherlands; lars.leijten@radboudumc.nl (L.L.);; 2 Radboudumc Center of Expertise Neuro-Oncology, 6525 GA Nijmegen, The Netherlands; 3 Department of Radiology and Nuclear Medicine, University Medical Center Utrecht, 3584 CX Utrecht, The Netherlands; 4 Department of Neurosurgery, Radboud University Medical Center, 6525 GA Nijmegen, The Netherlands; 5 Department of Radiation Oncology, Radboud University Medical Center, 6525 GA Nijmegen, The Netherlands; 6 Department of Neurology, Radboud University Medical Center, 6525 GA Nijmegen, The Netherlands; 7 Department of Radiology and Nuclear Medicine, Amsterdam University Medical Centers, Vrije Universiteit Amsterdam, 1100 DD Amsterdam, The Netherlands; 8 Department of Radiology & Nuclear Medicine, Erasmus MC, University Medical Center Rotterdam, 3015 GD Rotterdam, The Netherlands; 9 Brain Tumor Center, Erasmus MC Cancer Institute, 3015 GD Rotterdam, The Netherlands; 10 Medical Delta, 2629 JH Delft, The Netherlands

**Keywords:** molecular imaging–cancer, MR–perfusion, neuro-oncology, PET/CT, PET/MR

## Abstract

**Simple Summary:**

This meta-analysis provides a first head-to-head comparison of PET and perfusion weighted magnetic resonance imaging (PWI) in the surveillance of post-treatment gliomas in order to distinguish tumor progression (TP) from treatment-related abnormalities (TRA). Although various reviews have been published on the use of either PET or PWI in this setting, no meta-analysis to date provides a head-to-head comparison of both techniques. The findings of this paper illuminate the strengths and limitations of each technique and enable clinicians to take more evidence-based decisions in their daily practice with regard to the imaging surveillance of gliomas.

**Abstract:**

The post-treatment imaging surveillance of gliomas is challenged by distinguishing tumor progression (TP) from treatment-related abnormalities (TRA). Sophisticated imaging techniques, such as perfusion-weighted magnetic resonance imaging (MRI PWI) and positron-emission tomography (PET) with a variety of radiotracers, have been suggested as being more reliable than standard imaging for distinguishing TP from TRA. However, it remains unclear if any technique holds diagnostic superiority. This meta-analysis provides a head-to-head comparison of the diagnostic accuracy of the aforementioned imaging techniques. Systematic literature searches on the use of PWI and PET imaging techniques were carried out in PubMed, Embase, the Cochrane Library, ClinicalTrials.gov and the reference lists of relevant papers. After the extraction of data on imaging technique specifications and diagnostic accuracy, a meta-analysis was carried out. The quality of the included papers was assessed using the QUADAS-2 checklist. Nineteen articles, totaling 697 treated patients with glioma (431 males; mean age ± standard deviation 50.5 ± 5.1 years) were included. The investigated PWI techniques included dynamic susceptibility contrast (DSC), dynamic contrast enhancement (DCE) and arterial spin labeling (ASL). The PET-tracers studied concerned [S-methyl-^11^C]methionine, 2-deoxy-2-[^18^F]fluoro-D-glucose ([^18^F]FDG), O-(2-[^18^F]fluoroethyl)-L-tyrosine ([^18^F]FET) and 6-[^18^F]-fluoro-3,4-dihydroxy-L-phenylalanine ([^18^F]FDOPA). The meta-analysis of all data showed no diagnostic superior imaging technique. The included literature showed a low risk of bias. As no technique was found to be diagnostically superior, the local level of expertise is hypothesized to be the most important factor for diagnostically accurate results in post-treatment glioma patients regarding the distinction of TRA from TP.

## 1. Introduction

Diffuse infiltrating gliomas can have an astrocytic or an oligodendroglial origin—World Health Organization (WHO) grades 2–4, depending on the subtype—and have a high morbidity and mortality, even with the optimal treatment consisting of surgical resection and postoperative chemoradiotherapy [1]. This is mainly due to their (microscopic) infiltrative growth pattern and therapy-resistant glioma stem cells, leading to frequently observed posttreatment tumor progression (TP): the renewed occurrence or progression of enhancing areas within the remaining tumor or surgical bed on follow-up conventional magnetic resonance imaging (MRI). However, treatment-related abnormalities, (TRA) such as pseudoprogression and radiation necrosis, have almost identical characteristics on MRI [2], resulting in a diagnostic challenge [3,4]. Considering the fact that both entities require a vastly different therapeutic approach and are associated with significantly different outcomes, in recent years more sophisticated imaging techniques have been suggested to help distinguish TP from TRA [5,6,7]. These techniques include perfusion-weighted MRI (PWI) and positron emission tomography (PET) imaging.

The gold standard for diagnosing TP is a surgical resection, but a non-invasive technique is preferable. The three most frequently used techniques to perform PWI include arterial spin labeling (ASL), dynamic contrast enhanced (DCE) and dynamic susceptibility contrast (DSC) PWI. ASL PWI is based on blood as an endogenous arterial tracer, whereas DCE and DSC require the administration of an exogenous contrast agent [8]. The diagnostic capacity of each technique with regard to the differentiation of TP and TRA was recently meta-analyzed, showing excellent diagnostic accuracy for each technique [9]. Which of the different PWI techniques is superior remains a topic of debate, since the scientific literature provides encouraging evidence for each technique [10,11,12]. 

As PET imaging provides information on the metabolic status of glioma it is considered a valuable alternative method to distinguish TP from TRA [13]. A variety of PET tracers have been described in the literature, including 2-deoxy-2-[^18^F]fluoro-D-glucose ([^18^F]FDG), [S-methyl-^11^C]methionine ([^11^C]MET), O-(2-[^18^F]fluoroethyl)-L-tyrosine ([^18^F]FET) and 6-[^18^F]-fluoro-3,4-dihydroxy-L-phenylalanine ([^18^F]FDOPA) [5,14]. Although the use of radio-active labelled glucose (^18^F-FDG) for PET imaging is widespread in nuclear medicine, its clinical use in neuro-oncological diseases is limited because of the high physiological uptake of glucose in the cerebral and cerebellar cortex, resulting in a poor tumor-to-background ratio. On the other hand, glioma cells have a distinctly increased nutritional demand for amino acids to enable rapid proliferation and, unlike glucose, normal brain tissue has a low physiological uptake of amino acids, providing a superior tumor-to-background contrast. Positron-emitting nuclide labelled amino acids are therefore interesting probes for imaging gliomas in the pre- and post-treatment setting using PET [15]. The main amino acid tracers used for glioma imaging are [^11^C]MET, [^18^F]FET and [^18^F]FDOPA, all of which are taken up by the cell from the extracellular space via the Na+-independent system L-type amino acid transporter [16].

The diagnostic capacities of each technique have been summarized recently [17]. It has become apparent that the use of either PET or PWI allows for a more accurate differentiation between TRA and TP, although there is no consensus on the superiority of either technique or their complementary information. Therefore, more research is needed to identify the most optimal use. In particular, a direct head-to-head comparison of both techniques would contribute to this knowledge gap.

Therefore, the current systematic review and meta-analysis set out to provide an overview of the scientific literature that investigated the use of PET imaging and PWI in a head-to-head comparison to differentiate TRA from TP in patients with glioma. By only including articles in which patients underwent *both* PWI *and* PET imaging, we aimed to provide methodologically sound guidance for clinicians as well as policy makers regarding the use of PWI and/or PET imaging in the radiological follow-up of post-treatment glioma patients when in doubt as to whether a lesion reflects TP or TRA.

## 2. Materials and Methods

This study concerns a systematic literature review and meta-analysis of scientific literature on the use of PWI and/or PET imaging in the radiological follow-up of post-treatment glioma patients with a new contrast-enhancing lesion in order to distinguish TP and TRA.

The Preferred Reporting Items for Systematic Reviews and Meta-Analyses (PRISMA) guidelines were followed while conducting this review. PRISMA is an evidence-based minimum set of items for reporting in systematic reviews and meta-analyses. For more information on the PRISMA methodology and the subsequent steps, please see the PRISMA webpage (prisma-statement.org) (accessed on 5 April 2021).

### 2.1. Literature Search

A systematic literature search was carried out in PubMed, Embase and the Cochrane Library. The search strings can be found in Table 1. The databases were searched between 12 April 2021 and 10 May 2021. The literature search was updated on 8 August 2022. There were no restrictions in the search strategy. The literature search was carried out by two investigators independently (D.H. and L.L.).

### 2.2. Assessment of the Retrieved Articles

All the literature was assessed based on title and abstract to decide which articles were relevant for this study. This was carried out by two investigators independently (D.H. and L.L.). Discrepancies were resolved by discussion. If no consensus was reached, a third investigator (J.N.) was consulted to settle the argument.

Following screening of the title and abstract, a full-text analysis was carried out by the two investigators independently (D.H. and L.L.). The criteria for articles to be eligible for inclusion in this review were: (1) the use of PET-CT and/or PET-MRI with [^18^F]FDG, [^11^C]MET, [^18^F]FET or [^18^F]FDOPA as well as PWI (ASL, DCE and/or DSC) in the same patient; (2) the study population comprised patients diagnosed with adult-type diffuse glioma (WHO grades 2–4) after a neurosurgical resection and radiation therapy and/or chemotherapy and (3) the aim was to differentiate between TP and TRA. Articles needed to provide true positive, true negative, false positive and false negative (TP, TN, FP, FN, respectively) numbers, sensitivity and specificity for each imaging technique. The exclusion criteria comprised articles in which brain metastases were investigated and articles presenting no original data (e.g., reviews).

After inclusion, a data-extraction sheet constructed by D.H. and J.N. was used to extract the data from each included article. Data that were extracted concerned: (1) name of first author, (2) year of publication, (3) number of included patients, (4) sex distribution of the included patients, (5) mean/median age of the patients (with range and/or standard deviation), (6) grading of gliomas following the WHO guidelines, (7) number of lesions investigated, (8) type of PET system used (PET-CT or PET-MRI), (9) PET tracer and administered activity, (10) PWI technique, (11) TP, TN, FP, FN numbers, sensitivity and specificity for the investigated imaging techniques separately, (12) investigated parameter for PET imaging (e.g., tumor-brain ratio or standardized uptake value) and used cut-off value, (13) investigated parameters for each PWI technique (e.g., rCBV) and used cut-off value, (14) gold standard methodology and (15) final diagnosis of investigated lesions.

The methodological quality of the included articles was assessed using the QUADAS-2 checklist. This tool helps to evaluate the risk of bias and the applicability of primary diagnostic accuracy articles. This was carried out by two investigators independently (D.H. and L.L.). Discrepancies were resolved by discussion. If no consensus was reached, a third investigator (J.N.) was consulted to settle the argument.

### 2.3. Statistical Analysis

All data obtained from the individual articles were examined for completeness of observations and to avoid duplication. TN, TP, FN and FP were converted as the outcome state varied between the included articles. The outcome state for this meta-analysis was defined as TP; therefore a TN outcome referred to a patient in which TRA was present which was correctly identified by imaging. FP, on the other hand, comprised the situation in which a patient was found to have a TRA lesion although imaging identified the lesion as TP. The data were analyzed using RevMan (RevMan Version 5.4, Cochrane, https://training.cochrane.org/online-learning/core-software-cochrane-reviews/revman) (accessed on 17 May 2021). Random effects models were applied for calculating the pooled sensitivity and specificity. In addition, a Forest plot was constructed for each included study. A summary receiver operating characteristic curve (SROC) was plotted for each meta-analyzed technique. We chose to apply random effects models to enable a clinically relevant interpretation of the results. A significance level of 0.05 was chosen for all statistical tests. The meta-analysis yielded estimated pooled sensitivity and specificity values with 95% confidence intervals (95% CI). When the 95% CIs overlapped, the sensitivity/specificity between groups was deemed not statistically significantly different [18,19].

## 3. Results

### 3.1. Overview

**Figure 1:**
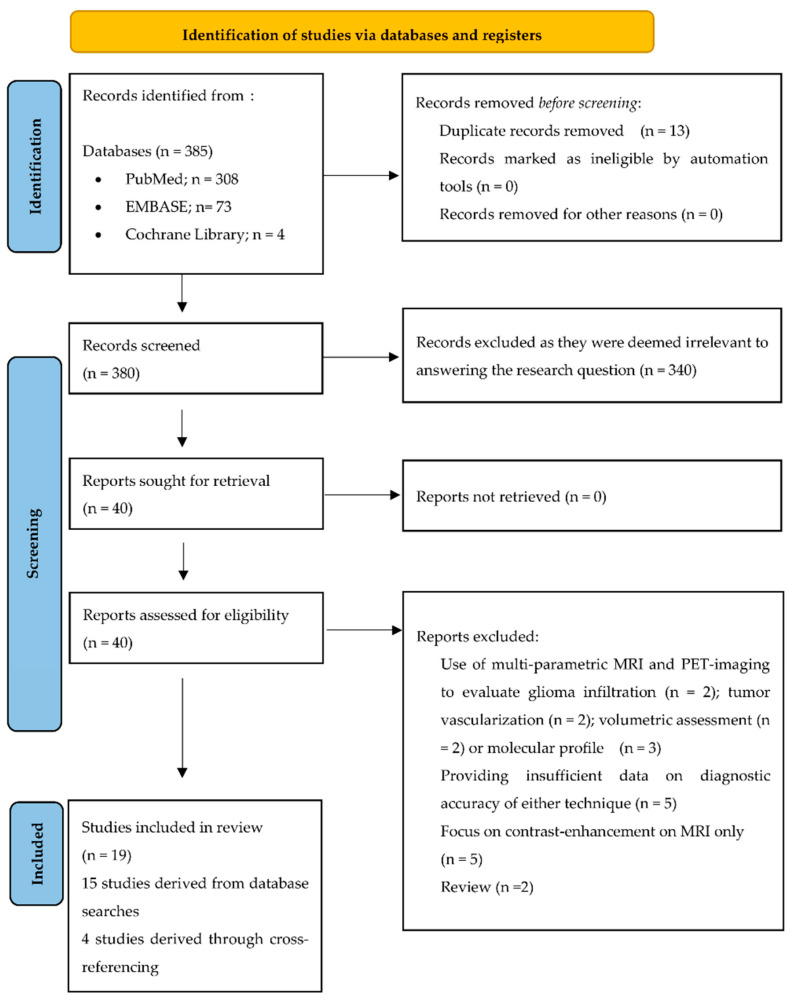
PRISMA flow chart.

Systematic searches yielded 385 articles and 8 articles were identified through cross-referencing. After the removal of all duplicates (*n* = 13), the remaining 380 articles were screened by title and abstract, leading to the exclusion of 340 articles, as they were deemed irrelevant for the purpose of this review. The full texts of the remaining 40 articles were analyzed (Figure 1). In total, 19 articles were included in this meta-analysis.

The 19 articles [20,21,22,23,24,25,26,27,28,29,30,31,32,33,34,35,36,37,38] were published between 2010 and 2022 and totaled 697 glioma patients (431 males) with a mean age ± standard deviation (SD) of 50.5 ± 5.1 years. The investigated PWI techniques included DSC PWI (14 articles) [20,21,22,23,24,25,26,31,32,33,34,35,36,38], DCE PWI (4 articles) [27,28,29,30] and ASL PWI (2 articles) [26,37]; the PET-tracers used included [^11^C]MET (5 articles) [20,21,22,23,38], [^18^F]FDG) (8 articles) [23,24,25,26,27,28,29,30], [^18^F]FET (5 articles) [31,32,33,34,35] and [^18^F]FDOPA (2 articles) [36,37]. A gold standard diagnosis was solely based on a histological assessment of brain tissue in three studies [20,21,26]. The assessed tissue in these studies was obtained by various neurosurgical interventions (i.e., brain biopsy or re-resection). In the remaining included studies, a gold standard diagnosis was based on a combination of radiological and clinical follow-up with or without the histological assessment of tissue after a brain biopsy. Again, when a histopathological assessment was carried out, the method of acquiring the brain tissue varied between and within studies (i.e., brain biopsy or re-resection). Table 2 provides an overview of the patient characteristics and imaging techniques of each study.

The methodological quality of the included articles is summarized in Table 3. Both retrospective and prospective articles were included in this meta-analysis. In most of the included articles, the investigators were not blinded to clinical and imaging information, which must be considered as a potential source of bias. In addition, none of the included articles described the use of a validation cohort to test the found cut-off values.

### 3.2. Meta-Analysis

Four subgroups could be distinguished: (1) papers in which [^11^C]MET PET was compared with DSC PWI (n = 5), (2) papers in which [^18^F]FDG PET was compared with DCE PWI (n = 4), (3) papers in which [^18^F]FDG PET was compared with DSC PWI (n = 4) and (4) papers in which [^18^F]FET PET was compared with DSC PWI (n = 5). The two papers on ASL PWI could not be meta-analyzed, as one paper compared ASL PWI with [^18^F]FDG PET, whereas the other paper focused on a comparison with [^18^F]FET PET. Additionally, the two papers on the use of [^18^F]FDOPA PET could also not be meta-analyzed as the PWI technique differed in each paper; one paper described the use of ASL PWI, whereas the other compared [^18^F]FDOPA PET with DSC PWI.

#### 3.2.1. [18F]FDG PET Imaging vs. DCE PWI

**Figure 2:**
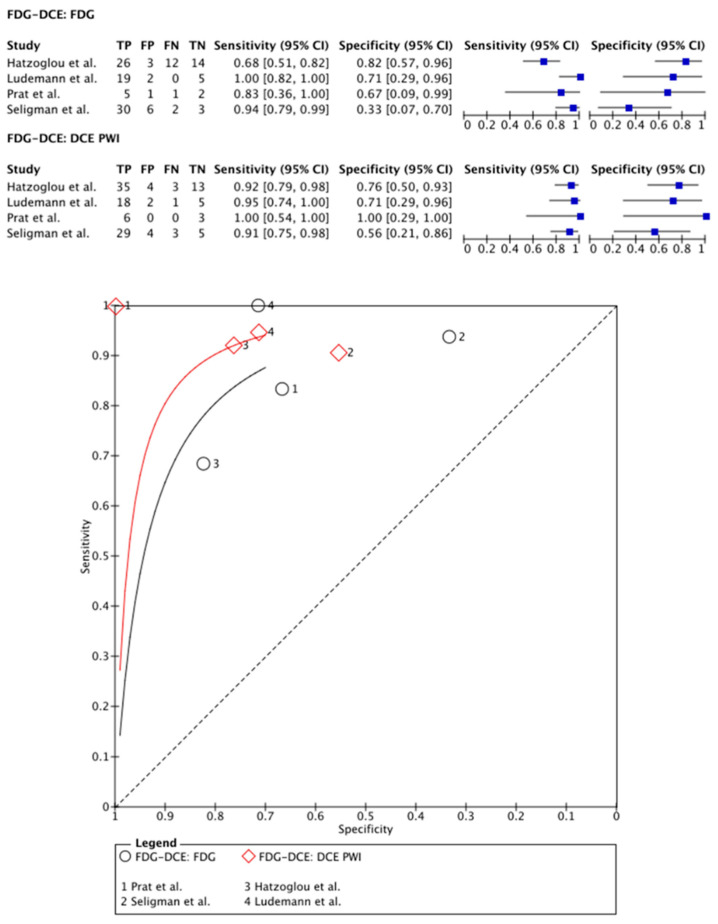
Forest plot and summary receiver operating curves (SROCs) of [^18^F]FDG PET imaging and DCE PW imaging with regard to the differentiation of TP from TRA. [^18^F]FDG, 2-deoxy-2-[^18^F]fluoro-D-glucose; DCE PW imaging, dynamic contrast enhancement perfusion weighted imaging; TP, tumor progression. Forest plot displaying the individual effect sizes from each study for the diagnostic accuracy of [^18^F]FDG PET imaging and DCE PW imaging with regard to the differentiation of TP from TRA. SROC of [^18^F]FDG PET imaging (black line) and DCE PW imaging (red line) with regard to the differentiation of TP from TRA shows greater potential for DCE PW imaging. However, the difference in the diagnostic accuracy of the techniques was not statistically significant [27,28,29,30].

[^18^F]FDG PET was compared with DCE PWI in four papers, totaling 112 patients (47 males and 24 females; the study of Seligman et al. [30] provided no information on sex distribution) [27,28,29,30]. In two papers, PET-CT systems were used for PET imaging and DCE PWI was performed separately [27,29]. Only the paper of Hatzoglou et al. describes the applied time interval: both imaging modalities were applied within a time period of 12 weeks [27]. In the other two articles, a hybrid PET-MRI system was used, which allowed for simultaneous imaging [28,30]. Two papers used a quantitative assessment for both the PET and the DCE images [28,29]. For the PET data, the remaining two articles used a TBR ratio [27,30]. On a meta-level, [^18^F]FDG PET showed a sensitivity of 89% (95% CI: 70–97%) and a specificity of 64% (95% CI: 40–83%), whereas DCE in these articles showed a sensitivity of 93% (95% CI: 85–96%) and a specificity of 72% (95% CI: 56–84%) (Figure 2).

#### 3.2.2. [^18^F]FDG PET Imaging vs. DSC PWI

**Figure 3:**
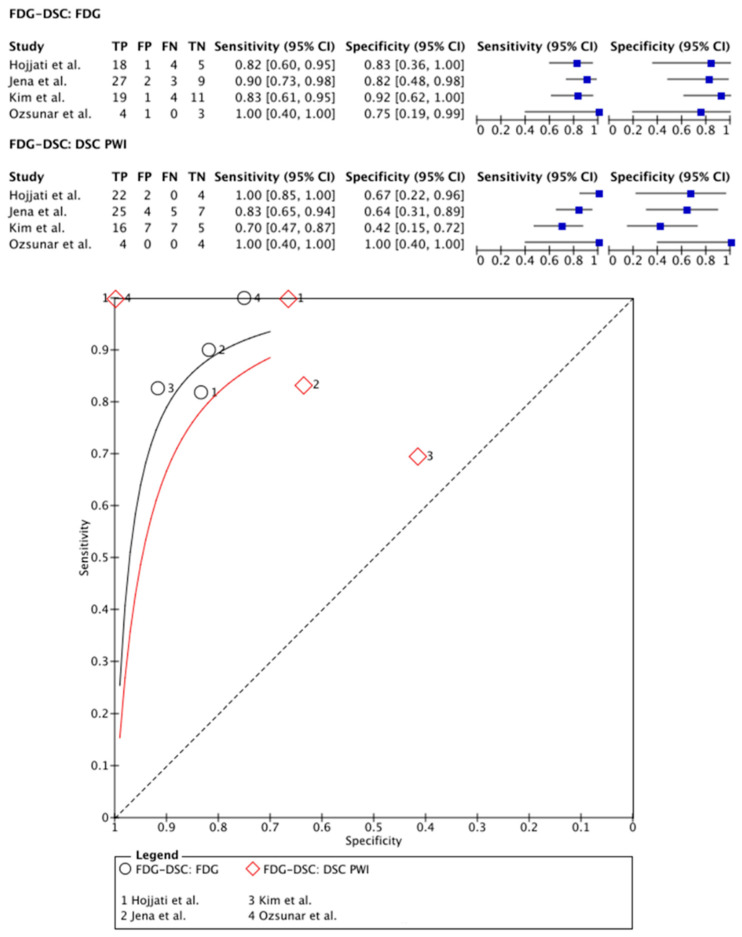
Forest plot and summary receiver operating curves (SROCs) of [^18^F]FDG PET imaging and DSC PW imaging with regard to the differentiation of TP from TRA. [^18^F]FDG, 2-deoxy-2-[^18^F]fluoro-D-glucose; DSC PW imaging, dynamic susceptibility contrast perfusion weighted imaging; TP, tumor progression. Forest plot displaying the individual effect sizes from each study for the diagnostic accuracy of [^18^F]FDG PET imaging and DSC PW imaging with regard to the differentiation of TP from TRA. SROC of [^18^F]FDG PET imaging (black line) and DSC PW imaging (red line) with regard to the differentiation of TP from TRA shows greater potential for [^18^F]FDG PET imaging. However, the difference in the diagnostic accuracy of the techniques was not statistically significant [24,25,26].

[^18^F]FDG PET was compared with DSC PWI in 99 patients (75 males) in four papers [23,24,25,26]. In two papers, PET-CT systems were used for PET imaging and DSC PWI was, therefore, performed separately within 0–30 days [23,26]. One study used a hybrid PET-MRI system, allowing for single-session imaging [25]. One paper compared the use of a PET-CT and hybrid PET-MRI system with a time frame of 10 min between PET-CT and PET-MRI imaging sessions (including DSC PWI sequences) [24]. Except for the paper of Ozsunar and colleagues [26], all papers analyzed their PET data using mean TBR [23,24,25]. Regarding the quantification of the DSC PWI data, all articles used rCBV parameters. On a meta-level, [^18^F]FDG PET showed a sensitivity of 86% (95% CI: 77–92%) and a specificity of 85% (95% CI: 66–94%), whereas DSC PWI in these articles showed a sensitivity of 92% (95% CI: 61–99%) and a specificity of 67% (95% CI: 40–87%) (Figure 3).

#### 3.2.3. [^11^C]MET PET Imaging vs. DSC PWI

In total, 157 patients (107 males) were included in the meta-analysis of [^11^C]MET PET imaging vs. DSC PWI as described in five papers [20,21,22,23,38]. In four papers, PET-CT systems were used for PET imaging and DSC PWI was performed separately within a period of 3–30 days [20,21,22,23]. One paper described the use of a hybrid PET-MRI system [38]. A variety of parameters were used to quantify the image analysis (i.e., uptake ratios comparing the left and right hemispheres and the tumor-brain ratio (TBR) max values) [21,22,23,38]. One paper analyzed PET images in a qualitative fashion [20]. For DSC PWI, all papers used the rCBVmax parameter for the image quantification analysis.

**Figure 4:**
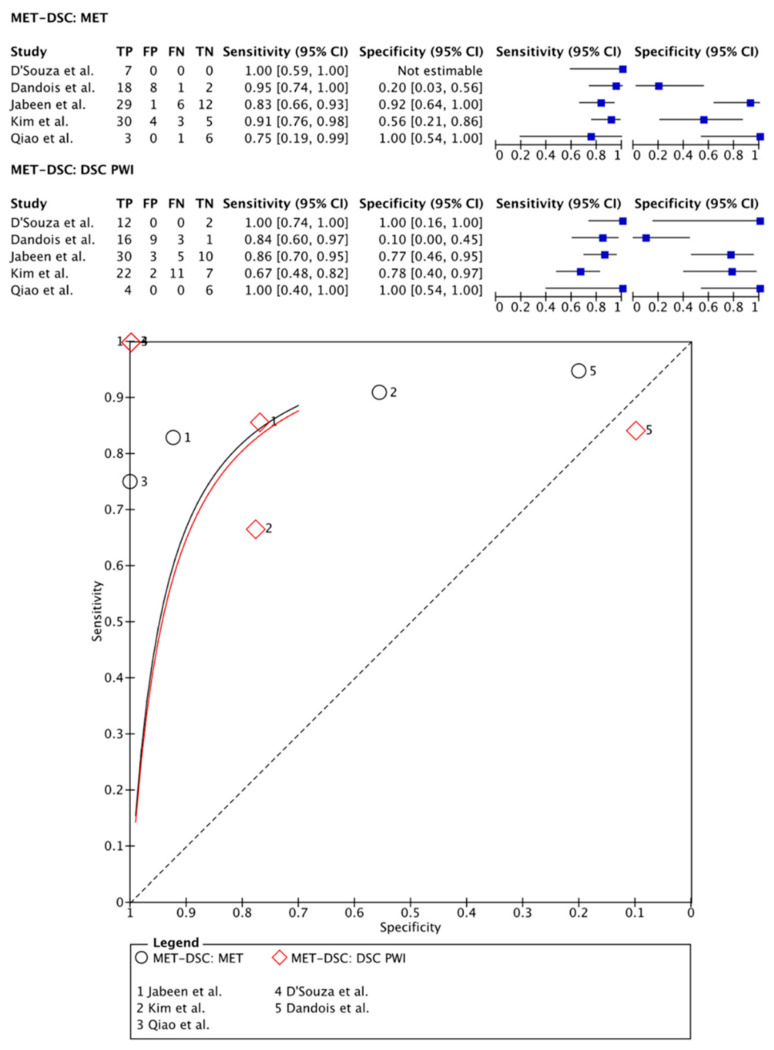
Forest plot and summary receiver operating curves (SROCs) of [^11^C]MET PET imaging and DSC PW imaging with regard to the differentiation of TP from TRA. [C_1_-11C]MET, [S-methyl-^11^C]methionine; DSC PW imaging, dynamic susceptibility contrast perfusion weighted imaging; TP, tumor progression. Forest plot displaying the individual effect sizes from each study for the diagnostic accuracy of [C_1_-11C]MET PET imaging and DSC PW imaging with regard to the differentiation of TP from TRA. SROC of [C_1_-11C]MET PET imaging (black line) and DSC PW imaging (red line) with regard to the differentiation of TP from TRA shows no superiority of either technique. Additionally, the difference in the diagnostic accuracy of the techniques was not statistically significant [20,21,22,23,38].

On a meta-level, [^11^C]MET PET showed a sensitivity of 89% (95% CI: 78–95%) and a specificity of 72% (95% CI: 25–95%), whereas DSC PWI in these articles showed a sensitivity of 90% (95% CI: 69–97%) and a specificity of 80% (95% CI: 27–98%) (Figure 4).

#### 3.2.4. [^18^F]FET PET Imaging vs. DSC PWI

**Figure 5:**
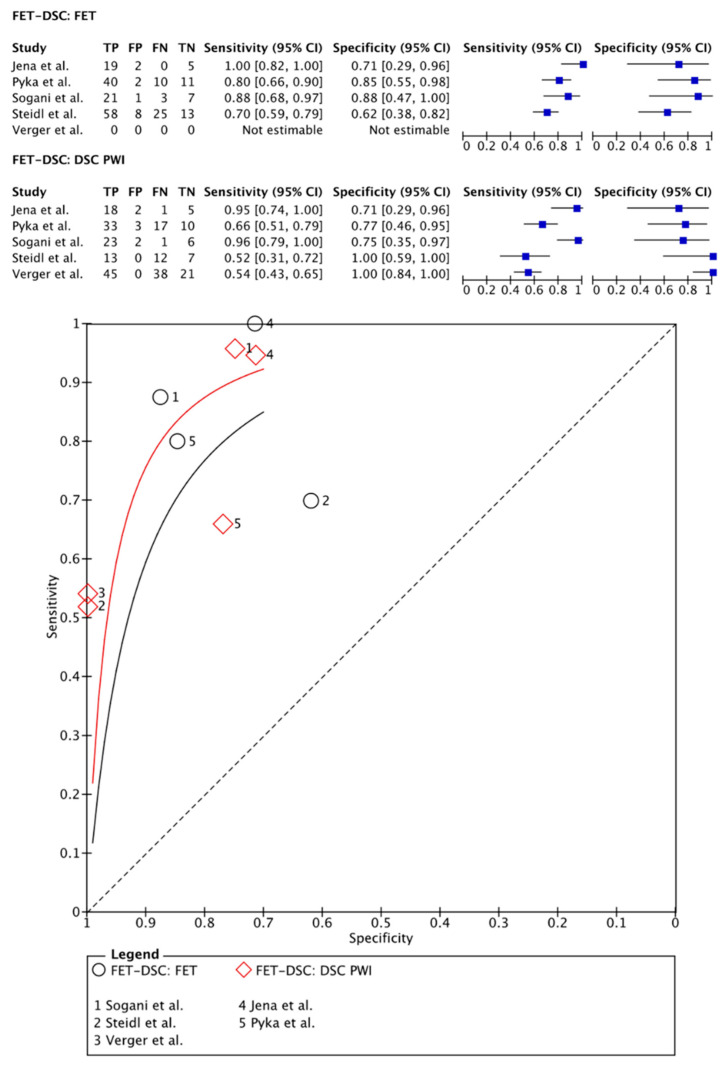
Forest plot and summary receiver operating curves (SROCs) of [^18^F]FET PET imaging and DSC PW imaging with regard to the differentiation of TP from TRA. [^18^F]FET, O-(2-[^18^F]fluoroethyl)-L-tyrosine; DSC PW imaging, dynamic susceptibility contrast perfusion weighted imaging; TP, tumor progression. Forest plot displaying the individual effect sizes from each study for the diagnostic accuracy of [^18^F]FET PET imaging and DSC PW imaging with regard to the differentiation of TP from TRA. SROC of [^18^F]FET PET imaging (black line) and DSC PW imaging (red line) with regard to the differentiation of TP from TRA shows greater potential for the DSC PW imaging. However, the difference in the diagnostic accuracy of the techniques was not statistically significant [31,32,33,34,35].

[^18^F]FET PET was compared with DSC PWI in 273 patients (178 males) in five papers [31,32,33,34,35]. One study used a PET-CT acquisition system and a separate MRI session with a maximum interval of 3 months between imaging sessions [35]. Other articles used a hybrid PET-MRI system allowing for simultaneous imaging [31,32,33,34]. Regarding the quantification of the PET images and the DSC PWI data, all articles used TBR parameters and rCBV parameters, respectively [31,32,33,34,35]. On a meta-level, [^18^F]FET PET showed a sensitivity of 82% (95% CI: 72–90%) and a specificity of 85% (95% CI: 68–94%), whereas DSC PWI in these articles showed a sensitivity of 76% (95% CI: 52–90%) and a specificity of 88% (95% CI: 67–96%) (Figure 5).

Given overlapping 95% CIs, the sensitivity values and most of the specificity values of the aforementioned grouped PET and PWI imaging methods, all techniques were considered comparable with regard to distinguishing TP from TRA in glioma patients. The pooled sensitivity and specificity values for each technique are provided in Table 4.

## 4. Discussion

This is the first meta-analysis of articles in which PWI and PET imaging were compared head-to-head in patients with treated glioma to distinguish TP from TRA, providing a unique insight into the diagnostic capacity of each technique. Despite inherent technological differences between PET imaging and PWI, the sensitivity and specificity were relatively comparable between these imaging methods when distinguishing TP from TRA in glioma patients. In addition, no significant differences were observed when comparing different PWI techniques and/or different PET tracers. Regarding the different PWI techniques, it has been reported that DSC PWI and DCE PWI provide similar diagnostic accuracy when distinguishing TP from TRA [39]. However, a more recent review by Van Dijken et al. showed higher diagnostic accuracy for DCE PWI compared with DSC PWI [40]. In terms of clinical relevance, this study shows that each imaging technique has comparable high pooled sensitivity rates with regard to diagnosing TRA. Thereby, a neurosurgical intervention (e.g., a brain biopsy) can be circumvented in a large number of cases when either of these imaging techniques is used.

PET imaging using either amino acid tracers or [^18^F]FDG showed no superiority/inferiority compared to the use of the different PWI techniques. Amino acid tracers, however, showed a higher sensitivity compared with [^18^F]FDG as a PET tracer. This agrees with previous publications on this topic [14] and can be explained by the poor TBR on [^18^F]FDG PET images, as [^18^F]FDG shows a high physiological uptake in the brain, complicating the detection of TP (no significant uptake) when a glucose-based PET tracer is used. Amino acid tracers, on the other hand, have a high TBR due to the significantly increased amino acid metabolism in glioblastoma cells to sustain cell proliferation and extracellular matrix production. In agreement with previously published reviews on the use of amino acid tracers and the detection of TP vs. TRA, there were no significant differences between the different amino acid radiotracers [14,17].

A major limitation in this field of research is that the cut-off values on which diagnostic accuracy (e.g., sensitivity, specificity) is based were determined without any form of internal or external validation. Thereby, there is a lack of robust data which limits the scientific evidence and hinders further standardization for future imaging trials. Additionally, in most of the included articles, readers were not blinded to the clinical/histopathological data and imaging information. Another limitation of the included articles concerns the variability in metrics which were meta-analyzed (e.g., rCBVmax, rCBVmean, ktransmean, TBRmean, TBRmax), especially in the [^18^F]FDG PET vs. DCE PWI meta-analysis. Nevertheless, when comparing TBRmax and TBRmean values to semiquantify FET data, Sogani et al. did not reveal significant differences regarding the diagnostic accuracy [33]. Therefore, it remains elusive to what extent this variability impacted the outcomes of the different diagnostic articles. In addition, as the reference standard to obtain the final diagnosis varied among studies, this could have partially influenced the results. In addition, the methods of obtaining brain tissue for histopathological examination varied within and between studies, which might have impacted the outcomes. Additionally, the QUADAS-2 checklist showed that histopathological criteria were not mentioned clearly by the authors. However, this was deemed as having only a minor impact on the outcomes of this review, as all papers referred to the use of the WHO Classification of Tumors of the Central Nervous System of either 2007 or 2016. Thereby, it was understood that histopathological diagnostics were carried out according to the then-applicable guidelines. Another limitation is the lack of harmonization of imaging protocols, which partially prohibits the discovery of robust imaging biomarkers needed for future research and clinical care. The European Imaging Biomarkers Alliance (EIBALL) and the Quantitative Imaging Biomarkers Alliance (QIBA), propelled by the European Society of Radiology and the Radiological Society of North America, respectively, collaborate to provide guidelines for future imaging articles. Furthermore, these societies aim to set standards for data acquisition, image processing and validation processes, as these three steps are essential for the development and implementation of imaging biomarkers in clinical trials and, ultimately, in the clinical setting [41,42].

Even though there are advantages of the additional use of PET over conventional MRI, the clinical implementation of PET-CT and PET-MRI techniques are limited by capacity, availability and logistical challenges, especially regarding the use of [^11^C]MET. PWI, on the other hand, is a widely available technique that has the additional advantage of being less expensive and less time-consuming than PET-CT or PET-MRI. Additionally, although their prevalence is continuously increasing, there are still only a few PET-MRI systems available in the world, limiting the use of simultaneous imaging protocols. Therefore, PWI is being implemented in a growing number of clinical practices. Based on the findings from this meta-analysis, we recommend performing routine radiological follow-up with the more widely available PWI. The high sensitivity rates of either PWI technique can help to exclude TP.

The combination of PET imaging and PWI has been suggested to further improve the overall diagnostic accuracy of the differentiation of TP from TRA [22,23,24,25,26,27,29,30,31,32,33,34]. A stepwise approach, where PWI-MRI is used to select cases for which PET imaging is most useful, can also increase diagnostic accuracy [35]. This could be valuable in more challenging cases with diagnostic uncertainties. The combination of exactly which PWI and PET technique is most optimal is not known and, therefore, should be determined by the level of local expertise. More prospective, blinded research is needed to investigate whether PET imaging indeed adds diagnostic value when PWI is inconclusive. It should be noted that most studies included different tumor types and that we could not perform meta-analyses per tumor type. The diagnostic accuracy for amino acid PET may increase for glioblastomas, as the use of [^18^F]FET PET imaging was shown to be significantly less accurate in IDH-mutant tumors compared to IDH-wildtype tumors in distinguishing TP from TRA [43].

Sub analyses per tumor type could provide important information on how the different techniques behave in different tumor types. Another factor that might influence diagnostic accuracy is the time period between radiotherapy and scan acquisition. For example, for amino acid PET the accuracy increases when image acquisition occurs at least 6 months after radiotherapy [44]. This factor is often not reported but may need more attention in future studies. Finally, it must be noted that the reference standard was different for different patients included in the analysis; some groups correlated their imaging findings with a histopathological assessment of obtained tissue, whereas the majority of articles described the use of clinical and radiological follow-up to determine the entity of an observed lesion.

More advanced analysis methods, including artificial intelligence methodologies, could be a promising solution for the near future. For example, a machine learning application was found to effectively distinguish TP from TRA on conventional MR imaging sequences alone [45,46,47]. The use of artificial intelligence applications on PW MR imaging data, however, has not been reported in the scientific literature. The application of artificial intelligence applications of PET-imaging, on the other hand, has been carried out by one group. Kebir et al. reported on the results of a preliminary study on the use of a machine learning model which used [^18^F]FET PET images to distinguish TP from TRA. In this study, they found that the applied machine learning approach had a significantly higher diagnostic accuracy with regard to differentiating TP from TRA when compared to the use of the TBRmax-value [48]. However, the aforementioned studies using artificial intelligence applications are limited by their use of relatively small datasets and the absence of an external validation dataset. These limitations should be addressed in future clinical trials using artificial intelligence applications to drive them to fully become powerful diagnostic tools in the future.

## 5. Conclusions

This meta-analysis demonstrates that PET imaging and PWI have similar diagnostic accuracy regarding the differentiation of TP from TRA in post-treatment glioma patients. Further research is necessary to optimize the complementary information provided by different imaging modalities for TRA and TP lesions, given that each modality uses distinct biological properties.

## Figures and Tables

**Table 1:** Search strings for different databases.

PubMed
*(“Glioma”[Title/Abstract] OR “glioblastoma”[Title/Abstract]) AND (“tumor recurrence”[Title/Abstract] OR “pseudoprogression”[Title/Abstract] OR “progression”[Title/Abstract]) AND (“PET”[Title/Abstract] OR “positron emission tomography”[Title/Abstract] OR “Positron Emission Tomography Computed Tomography”[MeSH Terms]) AND (“dynamic susceptibility contrast”[Title/Abstract] OR “dynamic contrast enhancement”[Title/Abstract] OR “arterial spin labeling”[Title/Abstract] OR (“Magnetic Resonance Imaging”[MeSH Terms] OR “Multiparametric Magnetic Resonance Imaging”[MeSH Terms] OR (“Magnetic Resonance Imaging”[Title/Abstract] OR “MRI”[Title/Abstract])))*
**EMBASE**
*(glioma or glioblastoma).m_titl.OR ((tumor recurrence or pseudoprogression or recurrence).mp. [mp = title, abstract, heading word, drug trade name, original title, device manufacturer, drug manufacturer, device trade name, keyword heading word, floating subheading word, candidate term word] OR (tumor recurrence or pseudoprogression or recurrence).m_titl.)) AND ((PET or positron emission tomography).mp. [mp = title, abstract, heading word, drug trade name, original title, device manufacturer, drug manufacturer, device trade name, keyword heading word, floating subheading word, candidate term word] OR (PET or positron emission tomography).m_titl) AND ((MRI perfusion.m_titl. OR MRI perfusion.mp. [mp = title, abstract, heading word, drug trade name, original title, device manufacturer, drug manufacturer, device trade name, keyword heading word, floating subheading word, candidate term word]) OR (dynamic contrast enhancement or dynamic susceptibility contrast or arterial spin labeling).m_titl. OR (dynamic contrast enhancement or dynamic susceptibility contrast or arterial spin labeling).mp. [mp = title, abstract, heading word, drug trade name, original title, device manufacturer, drug manufacturer, device trade name, keyword heading word, floating subheading word, candidate term word])*
**Cochrane Library**
*(((“glioblastoma”):ti,ab,kw OR (“glioma”):ti,ab,kw) OR ((“pseudoprogression”):ti,ab,kw OR (“tumor recurrence”):ti,ab,kw AND (“tumor progression”):ti,ab,kw)) AND (“positron emission tomography”):ti,ab,kw AND ((“perfusion weighted magnetic resonance imaging”):ti,ab,kw” OR (“dynamic susceptibility contrast”):ti,ab,kw OR (“dynamic contrast enhancement”):ti,ab,kw OR (“arterial spin labeling”):ti,ab,kw)*

**Table 2:** Overview of all included articles/studied populations.

*Study*	*Patients* *(n)*	*M/F (n)*	*Age (Years)*	*WHO Classification and Grade of Glioma*	*Lesions (n)*	*PET-Tracer*	*PET-CT* vs. *PET-MRI*	*Dose*	*Sens*	*Spec*	*PWI Technique*	*Sens*	*Spec*	*Lesion Diagnosis (Gold Standard)*	*Parameter PET*	*Cut-Off*	*Parameter PWI*	*Cut-Off*
*Dandois* et al. *(2010)* [20]	*28*	*16/12*	*mean 51 (range 25–74)*	*WHO grade 3 astrocytoma (9); WHO grade 3 oligodendroglioma (5); WHO grade 4 glioblastoma (14)*	*28*	*[^11^C]MET*	*PET-CT*	*740 MBq*	*100*	*N/E*	*DSC*	*67*	*100*	*Histological assessment after biopsy*	*Qualitative assessment*		*rCBV*	*>1.82*
*Kim* et al. *(2010); part 1* [23]	*10*	*8/2*	*mean age: 46.1 years*	*WHO grade 3 astrocytoma (3); WHO grade 3 oligodendroglioma (2); WHO grade 4 (5)*	*10*	*[^18^F]FDG*	*PET-CT*	*NR*	*100*	*75*	*DSC*	*100*	*100*	*Histological assessment after biopsy and/or radiological/clinical follow-up*	*Uptake ratios Lmax/Rmax*	*2.64*	*L/R ratio from rCBV*	*>3.69*
*Kim* et al. *(2010); part* *2* [23]	*10*	*8/2*	*mean age: 46.1 years*	*WHO grade 3 astrocytoma (3); WHO grade 3 oligodendroglioma (2); WHO grade 4 (5)*	*10*	*[^11^C]MET*	*PET-CT*	*NR*	*75*	*100*	*DSC*	*100*	*100*	*Histological assessment after biopsy and/or radiological/clinical follow-up*	*Uptake ratios Lmax/Rmax*	*2.64*	*L/R ratio from rCBV*	*>3.69*
*Ozsunar* et al. *(2010); part 1* [26]	*30*	*22/8*	*mean 42 (SD 11)*	*WHO grade 2 (7); WHO grade 3 (9); WHO grade 4 (19)*	*30*	*[^18^F]FDG*	*PET-CT*	*185–370 MBq*	*81*	*90*	*DSC*	*71*	*40*	*Histological assessment after biopsy*	*Qualitative assessment*		*normalized rCBV*	*>1.5*
*Ozsunar* et al. *(2010); part 2* [26]	*30*	*22/8*	*mean 42 (SD 11)*	*WHO grade 2 (7); WHO grade 3 (9); WHO grade 4 (19)*	*30*	*[^18^F]FDG*	*PET-CT*	*185–370 MBq*	*81*	*90*	*ASL*	*94*	*52*	*Histological assessment after biopsy*	*Qualitative assessment*		*normalized rCBV*	*>1.3*
*Prat* et al. *(2010)* [29]	*9*	*5/4*	*44.5 (16.3)*	*WHO grade 2 astrocytoma (3); WHO grade 2 oligodendroglioma (1); WHO grade 3 astrocytoma (5); WHO grade 3 oligodendroglioma (3); WHO grade 4 (11)*	*9*	*[^18^F]FDG*	*PET-CT*	*NR*	*83*	*67*	*DCE*	*100*	*100*	*Histological assessment after biopsy and/or radiological/clinical follow-up*	*Qualitative assessment*		*Qualitative assessment*	
*D’Souza* et al. *(2014)* [21]	*29*	*24/17*	*NR*	*WHO grade 3 astrocytoma (16); WHO grade 4 glioblastoma (13)*	*29*	*[^11^C]MET*	*PET-CT*	*6 MBq/kg*	*95*	*20*	*DSC*	*84*	*90*	*Histological assessment after biopsy*	*L/R ratio from SUVmean*	*>1.58*	*rCBV*	*>1.82*
*Hatzoglou* et al. *(2015)* [27]	*53*	*35/18*	*mean 57 (range 19–81)*	*WHO grade 2 astrocytoma (2); WHO grade 3 astrocytoma (6); WHO grade 2 oligodendroglioma (1); WHO grade 3 oligodendroglioma (2); WHO grade 4 glioblastoma (18); 24 metastases*	*29*	*[^18^F]FDG*	*PET-CT*	*370 MBq*	*68*	*82*	*DCE*	*92*	*77*	*Histological assessment after biopsy and/or radiological/clinical follow-up*	*SUV ratio*	*>1.2*	*Vp ratio*	*>2.1*
*Jena* et al. *(2016)* [31]	*26*	*21/5*	*mean 51.6 (SD 16.0)*	*NR*	*32*	*[^18^F]FET*	*PET-MRI*	*352.12 ± 64.26*	*100*	*71.4*	*DSC*	*96*	*71.4*	*Histological assessment after biopsy and/or radiological/clinical follow-up*	*TBRmax*	*>2.11*	*rCBV mean*	*>1.89*
*Jena* et al. *(2017)* [25]	*35*	*29/6*	*mean 50 (SD 12.0)*	*WHO grade 2 (9); WHO grade 3 (13); WHO grade 4 (19)*	*41*	*[^18^F]FDG*	*PET-MRI*	*222 ±* *30 MBq*	*90*	*81.8*	*DSC*	*83*	*63.6*	*Histological assessment after biopsy and/or radiological/clinical follow-up*	*TBRmean*	*>1.18*	*rCBVmean*	*>1.7*
*Sogani* et al. *(2017); part 1* [33]	*32*	*25/7*	*52.3 (17–80)*	*NR*	*32*	*[^18^F]FET*	*PET-MRI*	*207.2 ± 25 MBq*	*89*	*86,2*	*DSC*	*95*	*72*	*Histological assessment after biopsy and/or radiological/clinical follow-up*	*TBRmax*	*>2.09*	*rCBVmean*	*>1.78*
*Sogani* et al. *(2017); part 2* [33]	*32*	*25/7*	*52.3 (17–80)*	*NR*	*32*	*[^18^F]FET*	*PET-MRI*	*207.2 ± 25 MBq*	*89*	*86.2*	*DSC*	*95*	*72*	*Histological assessment after biopsy and/or radiological/clinical follow-up*	*TBRmean*	*>1.52*	*rCBVmean*	*>1.78*
*Hojjati* et al. *(2018); part 1* [24]	*24*	*16/8*	*mean 57.5 (range 34–81)*	*WHO grade 4 (24)*	*23*	*[^18^F]FDG*	*PET-MRI*	*440 MBq*	*100*	*80*	*DSC*	*100*	*75*	*Histological assessment after biopsy and/or radiological/clinical follow-up*	*TBRmean*	*>1.31*	*rCBVmax*	*>3.32*
*Hojjati* et al. *(2018); part 2* [24]	*24*	*16/8*	*mean 57.5 (range 34–81)*	*WHO grade 4 (24)*	*23*	*[^18^F]FDG*	*PET-CT*	*440 MBq*	*83*	*80*	*DSC*	*100*	*75*	*Histological assessment after biopsy and/or radiological/clinical follow-up*	*TBRmean*	*>1.47*	*rCBVmax*	*>3.32*
*Pyka* et al. *(2018)* [32]	*47*	*22/25*	*mean 54 (SD 11)*	*WHO grade 2 astrocytoma (2); WHO grade 2 oligodendroglioma (1); WHO grade 3 astrocytoma (13); WHO grade 3 oligodendroglioma (3); WHO grade 4 (27)*	*63*	*[^18^F]FET*	*PET-MRI*	*190 MBq*	*80*	*85*	*DSC*	*66*	*77*	*Histological assessment after biopsy and/or radiological/clinical follow-up*	*TBRmax*	*>2.07*	*rCBVmean*	*>3.35*
*Qiao* et al. *(2018)* [22]	*42*	*28/14*	*mean 47.2 (SD 10.5)*	*WHO grade 3 astrocytoma (12); WHO grade 3 oligodendroglioma (7); WHO grade 4 (23)*	*42*	*[^11^C]MET*	*PET-CT*	*370–738.8* *MBq*	*91*	*56*	*DSC*	*67*	*77.8*	*Histological assessment after biopsy and/or radiological/clinical follow-up*	*TBRmax*	*>1.85*	*rCBVmean*	*>1.83*
*Verger* et al. *(2018)* [34]	*32*	*17/15*	*mean age, 52 (SD 13.4)*	*WHO grade 2 astrocytoma (1); WHO grade 2 oligodendroglioma (1); WHO grade 3 astrocytoma (2); WHO grade 3 oligodendroglioma (1); WHO grade 4 (27)*	*32*	*[^18^F]FET*	*PET-MRI*	*3 MBq/kg*	*80*	*86*	*DSC*	*52*	*0*	*Histological assessment after biopsy and/or radiological/clinical follow-up*	*TBRmax*	*>2.61*	*rCBVmean*	*NR*
*Lundemann* et al. *(2019); part1* [28]	*9*	*7/2*	*mean 58.7 (SD 12.1)*	*WHO grade 4 (9)*	*9*	*[^18^F]FDG*	*PET-MRI*	*200 MBq*	*100*	*71.4*	*DCE*	*96*	*71.4*	*Histological assessment after biopsy and/or radiological/clinical follow-up*	*Qualitative assessment*		*Qualitative assessment*	
*Lundemann* et al. *(2019); part 2* [28]	*9*	*7/2*	*mean 58.7 (SD 12.1)*	*WHO grade 4 (9)*	*9*	*[^18^F]FET*	*PET-CT*	*200 MBq*	*90*	*81.8*	*DCE*	*83*	*63.6*	*Histological assessment after biopsy and/or radiological/clinical follow-up*	*Qualitative assessment*		*Qualitative assessment*	
*Seligman* et al. *(2019)* [30]	*41*	*NR*	*median 53 (21–79)*	*WHO grade 3 (21); WHO grade 4 (20)*	*41*	*[^18^F]FDG*	*PET-MRI*	*NR*	*94*	*33*	*DCE*	*91*	*56*	*Histological assessment after biopsy and/or radiological/clinical follow-up*	*TBRmean (Whole-tumor SUVmean divided by* *SUVmean of normal WM)*	*>0.75*	*Ktransmean (Mean Ktrans of whole tumor divided by mean Ktrans of contralateral brain)*	*>4.5*
*Fraioli* et al. *(2020)* [36]	*40*	*23/17*	*median 34 years (range 5–65)*	*WHO grade 1 (3); WHO grade 2 (12); WHO grade 3 (14); WHO grade 4 (11); glioblastoma (11); astrocytoma (23); oligodendroglioma (6)*	*40*	*[^18^F]* *FDOPA*	*PET-MRI*	*250–* *370 MBq*	*100*	*100*	*DSC*	*99*	*25*	*Histological assessment after biopsy and/or radiological/clinical follow-up*	*Qualitative assessment*		*Qualitative assessment*	
*Steidl* et al. *(2021)* [35]	*104*	*68/36*	*median age of 52 (range 20–78)*	*WHO grade 2 (9); WHO grade 3 (24); WHO grade 4 (71)*	*104*	*[^18^F]FET*	*PET-CT*	*3 MBq/kg*	*70*	*60*	*DSC*	*54*	*100*	*Histological assessment after biopsy and/or radiological/clinical follow-up*	*TBRmax*	*>1.95*	*rCBVmax*	*>2.85*
*Pellerin* et al. *(2021)* [37]	*58*	*34/24*	*mean age 53.1 ± 14.3*	*WHO grade 2 (10); WHO grade 3 (21); WHO grade 4 (27)*	*58*	*[^18^F]* *FDOPA*	*PET-MRI*	*2 MBq/kg*	*94.1*	*79.2*	*ASL*	*64.7*	*100*	*Histological assessment after biopsy and/or radiological/clinical follow-up*	*L/R 2 sample t-test*	*t > 6.36*	*L/R 2 sample t-test*	*t > 3.25*
*Jabeen* et al. *(2021)* [38]	*48*	*31/17*	*mean age 39.9 ± 12.5*	*WHO grade 2 (3); WHO grade 3 (28); WHO grade 4 (17)*	*48*	*[^11^C]MET*	*PET-MRI*	*360–378 MBq*	*81.8*	*92.3*	*DSC*	*84.8*	*76.9*	*Histological assessment after biopsy and/or radiological/clinical follow-up*	*TBRmax*	*>1.23*	*rCBVradio*	*>1.38*

[^18^F]FDG = 2-deoxy-2-[^18^F]fluoro-D-glucose; [^11^C]MET = [S-methyl-^11^C]methionine; [^18^F]FET = O-(2-[^18^F]fluoroethyl)-L-tyrosine; [^18^F]FDOPA = 6-[^18^F]-fluoro-3,4-dihydroxy-L-phenylalanine. When part 1 or part 2 is described, this indicates that the included paper comprises more than two groups relevant for meta-analysis.

**Table 3:** Overview of the quality of the included articles. Green = low risk of bias; red = high of bias; yellow = unclear risk of bias.

*Study*	*Prospective?*	*Patient and Treatment Characteristics Compared?*	*Adequately Described the Treatment Protocol?*	*Potentially Confounding Adjuvant Treatments?*	*Did the Study Avoid Inappropriate Exclusions?*	*Interval between the Completion of Treatment and Imaging Documented?*	*PET Imaging Results Interpreted without Knowledge of the Results of PW Imaging and Vice Versa*	*Post-Processing Techniques Reproducible as Described?*	*Did More than One Investigator Process the Imaging Data? Was There an Evaluation of Inter-Rater Reliability?*	*Were Histological Criteria Defined?*	*Reference Standard Adequately Defined When Pathology Was Unavailable?*	*Pathology Interpreted without Knowledge of the Results of PET/PW Imaging Outcomes?*	*Did All Patients Receive the Same Reference Test?*	*Were All Patients Included in the Analysis?*
*Dandois* et al. *(2010)* *[20]*	●	●	●	●	●	●	●	●	●	●	●	●	●	●
*D’Souza* et al. *(2014)* *[21]*	●	●	●	●	●	●	●	●	●	●	●	●	●	●
*Fraioli* et al. *(2020)* *[36]*	●	●	●	●	●	●	●	●	●	●	●	●	●	●
*Hatzoglou* et al. *(2015)* *[29]*	●	●	●	●	●	●	●	●	●	●	●	●	●	●
*Hojjati* et al. *(2018); part1* *[24]*	●	●	●	●	●	●	●	●	●	●	●	●	●	●
*Hojjati* et al. *(2018); part2* *[24]*	●	●	●	●	●	●	●	●	●	●	●	●	●	●
*Jena* et al. *(2016)* *[31]*	●	●	●	●	●	●	●	●	●	●	●	●	●	●
*Jena* et al. *(2017)* *[25]*	●	●	●	●	●	●	●	●	●	●	●	●	●	●
*Lundemann* et al. *(2019); part1* *[28]*	●	●	●	●	●	●	●	●	●	●	●	●	●	●
*Lundemann* et al. *(2019); part 2* *[28]*	●	●	●	●	●	●	●	●	●	●	●	●	●	●
*Ozsunar* et al. *(2010); part 1* *[26]*	●	●	●	●	●	●	●	●	●	●	●	●	●	●
*Ozsunar* et al. *(2010); part 2* *[26]*	●	●	●	●	●	●	●	●	●	●	●	●	●	●
*Pyka* et al. *(2018)* *[32]*	●	●	●	●	●	●	●	●	●	●	●	●	●	●
*Qiao* et al. *(2018)* *[22]*	●	●	●	●	●	●	●	●	●	●	●	●	●	●
*Sogani* et al. *(2017); part 1* *[33]*	●	●	●	●	●	●	●	●	●	●	●	●	●	●
*Sogani* et al. *(2017); part 2* *[33]*	●	●	●	●	●	●	●	●	●	●	●	●	●	●
*Kim* et al. *(2010); part 1* *[23]*	●	●	●	●	●	●	●	●	●	●	●	●	●	●
*Kim* et al. *(2010); part 2* *[23]*	●	●	●	●	●	●	●	●	●	●	●	●	●	●
*Prat* et al. *(2010)* *[29]*	●	●	●	●	●	●	●	●	●	●	●	●	●	●
*Seligman* et al. *(2019)* *[28]*	●	●	●	●	●	●	●	●	●	●	●	●	●	●
*Verger* et al. *(2018)* *[34]*	●	●	●	●	●	●	●	●	●	●	●	●	●	●
*Steidl* et al. *(2021)* *[35]*	●	●	●	●	●	●	●	●	●	●	●	●	●	●
*Pellerin* et al. *(2021)* *[37]*	●	●	●	●	●	●	●	●	●	●	●	●	●	●
*Jabeen* et al. *(2021)* *[38]*	●	●	●	●	●	●	●	●	●	●	●	●	●	●

**Table 4:** Pooled sensitivity and specificity of each technique included in this review.

Technique	References	Patients (n)	Pooled Sensitivity	95% CI	Pooled Specificity	95% CI
DCE PWI	[27,28,29,30]	112	90%	84–94%	70%	56–82%
DSC PWI	[20,21,22,23,24,25,26,31,32,33,34,35,36,38]	497	90%	80–95%	77%	61–88%
ASL PWI	[26,37]	56	84%	31–98%	85%	11–100%
[^18^F]FDG PET	[23,24,25,26,27,28,29,30]	192	89%	80–94%	78%	65–87%
[^11^C]MET PET	[20,21,22,23,38]	157	89%	78–95%	72%	25–95%
[^18^F]FET PET	[28,31,32,33,34,35]	250	84%	75–90%	80%	67–88%
[^18^F]FDOPA	[36,37]	98	94%	86–98%	78%	58–90%

[^18^F]FDG, 2-deoxy-2-[^18^F]fluoro-D-glucose; [^11^C]MET, [S-methyl-^11^C]methionine; [^18^F]FET, O-(2-[^18^F]fluoroethyl)-L-tyrosine; [^18^F]FDOPA, 6-[^18^F]-fluoro-3,4-dihydroxy-L-phenylalanine.
